# Anemia in severe heart failure patients: does it predict prognosis?

**DOI:** 10.1186/s12872-017-0680-5

**Published:** 2017-09-16

**Authors:** Tamrat Befekadu Abebe, Eyob Alemayehu Gebreyohannes, Akshaya Srikanth Bhagavathula, Yonas Getaye Tefera, Tadesse Melaku Abegaz

**Affiliations:** 1 Department of Clinical Pharmacy, School of Pharmacy, College of Medicine and Health Sciences, University of Gondar, Gondar, Ethiopia; 2 Master’s program in Health Economics, Policy and Management, Student, Department of Learning Informatics, Management and Ethics (LIME), Karolinska Institutet, Solna, Sweden

**Keywords:** Heart Failure, Hemoglobin, Medical records, Survival, Mortality, Prognosis, Ethiopia

## Abstract

**Background:**

Anemia is highly prevalent in heart failure (HF) patients. However, the prevalence, clinical impact and prognostic factor of anemia in heart failure patients is widely varies. The aim of this study was to evaluate the prevalence of anemia in patients with HF, to compare baseline clinical characteristic and outcomes of severe HF patients with and without anemia admitted to Gondar University Referral Hospital (GURH), Gondar, Ethiopia.

**Method:**

A retrospective cohort study was conducted and we assessed medical records of heart failure patients who were admitted Gondar University Referral Hospital in the period between December 02, 2010 and November 30, 2016. Kaplan Meier curve was used to analyze the survival status and log rank test was used to compare the curves. Multivariate Cox regression was used to analyze independent predictors of mortality in all HF patients. *P* value less than 0.05 was considered statistically significant.

**Result:**

Three hundred and seventy patients participated in the study. The prevalence of anemia in the study cohorts was 41.90% and majority of the participants were females (64.59%). There was a significant difference in the level of hemoglobin, creatinine, and sodium among anemic and non-anemic patients. Anemic patients with HF tend to take angiotensin converting enzyme inhibitors (ACEI) less frequently. Kaplan Meier survival curves and Log rank test (*P* = 0.042) showed a significant difference in the prognosis of HF patients with anemia and non – anemic. More significant difference was observed (Log rank test, *P* = 0.001) in the study participants based on hemoglobin level. Furthermore, multivariate Cox regression showed: advanced age, levels of lower sodium and higher creatinine, and absences of medications like ACEI and Spironolactone independently predicted overall mortality.

**Conclusion:**

HF patients with anemia tend to be older age, had lower hemoglobin and sodium level and higher creatinine value. Moreover, there was a significant difference in the prognosis between study cohorts, as anemic pateints tend to have a worse survival status . Even though, anemia is a significant risk marker, it is not an independent predictor of mortality in the current study.

## Background

Anemia is highly prevalent in heart failure (HF) patients. Its prevalence among patients with HF estimates can range from 30% to 70% in some studies depending on the cutoff value used to define its presence and on the population considered [1, 2]. Even though anemia is a commonly observed condition in HF, associated with significantly worse prognosis, there is no certain explanation, how it affects mortality, provokes HF exacerbation and influences the course of hospitalization [3–8]. Some of the proposed mechanism for anemia in HF include: iron deficiency due to reduced intestinal absorption or cytokine-related inflammatory changes, reduced erythropoietin production, simultaneous comorbidities such as renal failure, or even hemodilution [6, 9, 10]. The prevalence of anemia and its impact on patient’s survival outcome among reduced (HFrEF) and preserved (HFpEF) ejection fraction appears to be comparable, with comparable risks of mortality, readmission, hospitalization rates and loss of functional capacity [11, 12]. There are encouraging reports on the effectiveness of iron therapy in reducing HF symptoms, however, there are still no favorable results in terms of mortality in HF [13–16]. Thus, establishing the clinical importance of anemia in HF patients is imperative.

In Ethiopia, there is no study which demonstrated the overall prevalence of anemia in HF patients at national level; however, there are some studies which highlighted its prevalence in cardiovascular patients at institutional level to be between 3 and 25% depending on the diagnosis [17, 18].

The aim of this study was to evaluate the prevalence of anemia in patients with HF, to compare baseline clinical characteristic and outcomes of severe HF patients with and without anemia admitted to Gondar University Referral Hospital (GURH).

## Methods

This study is adapted from a previous study conducted by Abebe et al. [18]. The study design and preliminary results were published previously [18]. Patients who had been admitted to GURH Internal Medicine department with the diagnosis of HF in the period from December 02, 2010 to November 30, 2016 were assessed retrospectively using their medical records. Patients who had been diagnosed HF, are 18 years of age or older, and met the adapted Framingham criteria for the diagnosis of heart failure were included [19].

### Exclusion criteria were patients

- Who had infection in addition to HF on admission,
- Who did not have full laboratory and echocardiography data in their medical records and,
- Who were not symptomatic on admission (New York Heart Association (NYHA) Class I and class II) as adapted from a study conducted by Mozaffarian et al. [20].

Based on the above criteria 370 patients met the inclusion criteria from 980 patients who were admitted during the study period.

Per hemoglobin level on hospital admission, patients were categorized into anemic and non-anemic groups. Hemoglobin concentration of less than 13 g/dl for male and less than 12 g/dl for female was used to define anemia according to the world health organization (WHO) criteria [21].

During the first admission to the internal medicine ward, patient’s ejection fraction was measured by a radiologist using echocardiography. Last hospital discharge or medication refill time was used as a vital status to assess study participants’ survival status. Definitions for etiologies of HF were taken from a previous study by Abebe et al. [18]. Hypertension was determined as blood pressure 140/90 mmHg or more.

### Statistical analysis

Statistical analysis was carried out using the Statistical Package for Social Science, version 20.0 for Windows (SPSS, Chicago, IL, USA). Continuous variables were revealed as mean ± standard deviation and median (IQR) and discrete variables presented as percentage. Prior to additional analyses, Shapiro – Wilk and Levene test was performed to assess the data for normality and homogeneity. Patients were categorized based on their anemia status and further analysis was conducted using student t – test for Continuous variables and chi – square test for discrete variables to assess baseline characteristics, laboratory and echocardiography results and medication prescription among the study groups. Kaplan-Meier survival analysis was conducted to measure event free survival and the Mantel Log – rank test for between groups comparison. Cox proportional hazard ratio was used for the univariate analysis of predictor of events. The variables that had *P* values less than 0.2 in the univariate analysis were included in the Cox multivariate analysis. Hazard ratio and 95% confidence interval were shown. A type I error with *P* value less than 0.05 was considered significant.

During the study, patient’s data was de – identified to protect anonymity of medical records.

## Result

Of 980 patients who were admitted to GURH due to HF, in the period between December 02, 2010 and November 30, 2016, 370 patients met the inclusion criteria. From the study group, 155 patients had anemia and the remaining participants (215) were non-anemic. Table 1 shows baseline clinical characteristics of the two groups. The mean age of the participants was 54.56 (±17.49) years with significant difference among the groups. There was no significant disparity among HF patients with and without anemia, hypertension, or AF, based on NYHA class, heart rate, or BP. Also, there was no difference between the two groups based on etiology of HF except for cor pulmonale (8.84% Vs. 0.65%, *P* = < 0.001).

**Table 1:** Clinical characteristics of heart failure patients based on anemia status

Variable		Non – anemic (215)	Anemic (155)	*P* – value
Age, mean ± SD		52.99 ± 17.15	56.47 ± 17.76	0.041*
Gender, n (%)				0.117
	Male	69 (32.09%)	62 (40.00%)	
	Female	146 (67.91%)	93 (60.00%)	
Residency, n (%)				0.075
	Urban	99 (46.05%)	57 (36.77%)	
	Rural	116 (53.95%)	98 (63.23%)	
NYHA Class, n (%)				0.339
	Class III	55 (25.58%)	33 (21.29%)	
	Class IV	160 (74.42%)	122 (78.71%)	
Hypertension, n (%)		63 (29.30%)	54 (34.84%)	0.258
AF, n (%)		51 (23.72%)	42 (27.10%)	0.460
Heart Rate, mean ± SD		91.06 ± 19.11	94.99 ± 19.64	0.056
Systolic BP, mean ± SD		120.66 ± 22.29	123.74 ± 25.60	0.219
Diastolic BP, mean ± SD		79.00 ± 14.30	79.50 ± 15.56	0.751
Etiology of HF, n (%)				
	IHD	36 (16.74%)	22 (14.19%)	0.506
	VHD	82 (38.14%)	74 (47.74%)	0.065
	HHD	33 (15.35%)	24 (15.48%)	0.972
	DCMP	25 (11.63%)	21 (13.55%)	0.581
	CorPulmonale	19 (8.84%)	1 (0.65%)	<0.001*
	Other etiology	20 (9.30%)	13 (8.34%)	0.761

*AF* Atrial Fibrillation, *BP* Blood Pressure, *DCMP* Dilated Cardiomyopathy, *HF* Heart Failure, *HHD* Hypertensive Heart Disease, *IHD* Ischemic Heart Disease, *NYHA* New York Heart Association, *SD* Standard Deviation, *VHD* Valvular Heart Disease

*p < 0.05

### Results of laboratory analysis and echocardiograms

Per Table 2, higher hemoglobin (14.32 ± 2.16 Vs. 10.11 ± 2.17, *P* = < 0.0001), greater sodium (135.79 ± 6.48 Vs. 133.20 ± 6.18, *P* = < 0.0001) and lower creatinine (0.99 ± 0.50 Vs. 1.33 ± 1.02, *P* = < 0.0001) levels were measured in HF patients with non-anemic than anemic, respectively.

**Table 2:** Laboratory and echocardiography results of heart failure patients based on anemia status

Variable	Non – anemic (215)	Anemic (155)	*P* – value
Hemoglobin (mean ± SD)	14.32 ± 2.16	10.11 ± 2.17	<0.0001*
Creatinine (mean ± SD)	0.99 ± 0.50	1.33 ± 1.02	<0.0001*
Sodium (mean ± SD)	135.79 ± 6.48	133.20 ± 6.18	<0.0001*
LVEF (mean ± SD)	52.56 ± 13.73	51.41 ± 13.75	0.425
LVEDD in mm (mean ± SD)	50.86 ± 10.87	51.92 ± 10.39	0.352

*LVEF* left ventricular ejection fraction, *LVEDD* left ventricular end diastolic dimension, *SD* standard deviation

*p < 0.05

### Medical treatment

There was no significant disparity in the medication profile between the study groups except for angiotensin converting enzyme inhibitors (ACEIs) where they were more frequently prescribed in the non-anemic groups (52.56% Vs. 41.94%, *P* = 0.044) (Table 3).

**Table 3:** Medication profile of heart failure patients based on anemia status

Variable	Non – anemic (215)	Anemic (155)	*P* – value
Diuretics n (%)	199 (92.56%)	135 (87.10%)	0.080
Spironolactone n (%)	148 (68.84%)	100 (64.52%)	0.383
ACEI n (%)	113 (52.56%)	65 (41.94%)	0.044*
Beta Blocker n (%)	105 (48.84%)	77 (49.68%)	0.873
Digoxin n (%)	49 (22.79%)	43 (27.74%)	0.277
CCB n (%)	14 (6.51%)	17 (10.97%)	0.127
Antiplatelet n (%)	45 (20.93%)	36 (23.23%)	0.598
Anticoagulants n (%)	48 (30.97%)	33 (21.29%)	0.812
Statin n (%)	34 (15.81%)	27 (17.42%)	0.681

*ACEI* Angiotensin converting enzyme inhibitor, *CCB* calcium channel blocker

*p < 0.05

### Survival analysis

**Fig. 1:**
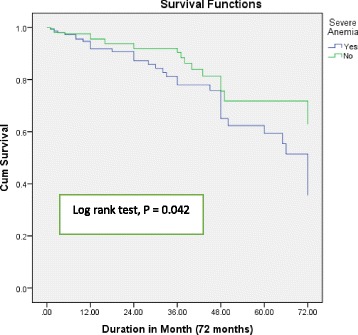
Kaplan Meier curves free from all causes of death in Heart Failure Patients based on anemia status

**Fig. 2:**
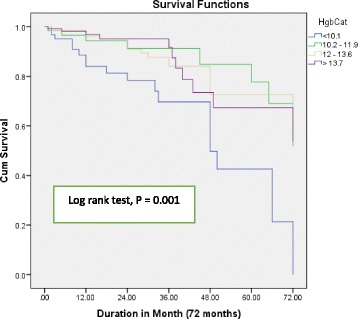
Kaplan Meier curves free from all causes of death in Heart Failure Patients based on hemoglobin status. Hgb Cat: Hemoglobin Category

The median (IQR) duration of follow up was 18(8 - 36) months. Mortality were 21.94% in HF patients with anemia (34 patients) and 9.78% in patients without anemia (21 patients). Kaplan Meier survival curves (Fig. 1) shows that there was significant difference in survival status of HF patients with and without anemia (Log Rank test, *P* = 0.042). Furthermore, a stronger significance differences (Log rank test, *P* = 0.001) were observed in the survival status of patients with HF when study participants were grouped based on their hemoglobin value (< 10.1 g/dl; 10.2 – 11.9 g/dl; 12–13.6 g/dl and ≥ 13.7 g/dl) (Fig. 2). On the other hand, univariate cox regression analysis showed that age, systolic blood pressure, diastolic pressure, Ischemic heart disease, anemia, creatinine level, sodium level, left ventricular ejection fraction, and use of spironolactone, ACEIs, beta blockers and anticoagulants were significantly associated with mortality in patients with HF. Multivariate cox regression analysis, showed that advanced age, lower sodium value, higher creatinine level and absence of medication use such as ACEI and spironolactone significantly predicted mortality in HF patients (Table 4). Even though, anemia is a significant risk marker, it is not an independent predictor of mortality in the current study.

**Table 4:** Predictors of mortality to all causes of heart failure

Variables		Univariate analysis	Multivariate analysis	
		Hazard ratio (95% CI)	Hazard ratio (95% CI)	*P*- value
Gender	Female	1.122 (0.643–1.958)	–	
Age, Years		1.039 (1.021–1.058)	1.041 (1.017–1.066)	0.001*
NYHA Class	Class IV	1.622 (0.764–3.442)	0.946 (0.402–2.227)	0.899
Heart Rate, bpm		1.005 (0.992–1.017)	–	
Systolic BP, mmHg		1.017 (1.008–1.026)	0.992 (0.975–1.009)	0.367
Diastolic BP, mmHg		1.031 (1.016–1.046)	1.020 (0.997–1.044)	0.089
Anemia		1.740 (1.004–3.016)	0.862 (0.439–1.693)	0.666
Sodium, mEq/L		0.937 (0.903–0.973)	0.933 (0.882–0.987)	0.016*
Creatinine, mg/dl		1.877 (1.636–2.155)	1.869 (1.460–2.392)	<0.0001*
AF	Yes	0.535 (0.275–1.039)	1.913 (0.583–6.272)	0.284
VHD	Yes	0.635 (0.366–1.103)	0.821 (0.418–1.612)	0.566
IHD	Yes	2.261 (1.228–4.162)	0.620 (0.256–1.501)	0.289
HHD	Yes	0.918 (0.449–1.876)	–	
DCMP	Yes	0.878 (0.346–2.227)	–	
Cor pulmonare	Yes	0.776 (0.106–5.691)	–	
Other etiology	Yes	1.237 (0.443–3.453)	–	
LVEF, %		0.977 (0.955–0.998)	0.984 (0.955–1.014)	0.283
Diuretics	Yes	0.586 (0.229–1.496)	–	
Spironolactone	Yes	0.359 (0.210–0.613)	0.527 (0.276–0.996)	0.050*
ACEI	Yes	0.546 (0.311–0.960)	0.410 (0.197–0.852)	0.017*
Beta Blocker	Yes	0.555 (0.320–0.964)	0.578 (0.295–1.132)	0.110
Digoxin	Yes	0.401 (0.201–0.800)	0.634 (0.225–1.791)	0.390
Antiplatelates	Yes	0.895 (0.478–1.674)	–	
Anticoagulants	Yes	0.400 (0.180–0.888)	0.841 (0.277–2.558)	0.760
Statin	Yes	1.431 (0.754–2.715)	–	
CCB	Yes	1.524 (0744–3.119)	–	

*ACEI* Angiotensin converting enzyme inhibitor, *AF* Atrial Fibrillation, *BP* blood pressure, *CCB* calcium channel blocker, *DCMP* Dilated Cardiomyopathy, *HHD* hypertensive heart disease, *IHD* Ischemic heart disease, *LVEF* Left ventricular ejection fraction, *NYHA* New York Heart Association

*p < 0.05

## Discussion

Anemia has lately been renowned as an imperative comorbidity and potential novel therapeutic target in heart failure patients [5]. To our knowledge, this study is the first to identify both the prevalence of anemia and its influence on survival status in patients with heart failure in Ethiopia. A meta-analysis conducted by Groenveld HF et al. reported the prevalence of anemia as 37.2% in patients with HF [5]. Furthermore, in a study of anemia in a population with heart failure (STAMINAHFP), the prevalence of anemia was 34% among outpatients with chronic heart failure, based on the WHO criteria for anemia [22]. In the current study, the prevalence of anemia was around 41.90% in HF patients. Our finding was higher than previous studies. This was attributable to patients’ characteristics such as gender, age, use of inconsistent definitions for anemia in patients with heart failure [23, 24] and inclusion of severely anemic patients in the study; unlike in most randomized clinical trials, severe anemia is an exclusion criterion, which makes it difficult to precisely assess this group of patients [25, 26].

In this study, age is significantly related with anemia with HF patients, which is a significant factor in most studies [27–29]. The mean age of anemic cohort in this study was (56.47 ± 17.76 years) which is younger than patients in the Swedish HF registry [30], the EVEREST trial [31], Valsartan heart failure (Val – HeFT) trial [25] and in IN – CHF registry [32]. This disparity might be due to the relative low sample size and younger population of the current study. Moreover, higher creatinine level, lower sodium and hemoglobin levels were significantly related with HF patients with anemia; these findings were in alignment with various studies [27–29, 33]. These all are substantially related with renal dysfunction, as glomerular filtration declines fluid volume in our body rises (hyponatremia) and creatinine clearance will decline. Moreover, renal dysfunction will result decreased erythropoietin synthesis which will affect red blood cell production and hemoglobin concentration [28, 33].

In the current study, left ventricular ejection fraction (LVEF) was not associated with degree of anemia. However, certain studies illustrated that the prevalence of anemia among patients with preserved left ventricular ejection fraction and among those with reduced ejection fraction is comparable [34, 35]. In an analysis of the Candesartan in Heart Failure Assessment of Reduction in Mortality and Morbidity program (CHARM), lower Hemoglobin levels was associated with higher LVEF [33]. However, in a study of patients with restricted to impaired LVEF in the Valsartan Heart Failure Trial, the association between Hemoglobin and LVEF was not clear [25]. Further studies are required to clearly identify the association between anemia and LVEF.

Anemic patients are usually less likely to receive HF management according to guideline recommendations including ACEI, beta – blocker and Aldosterone antagonist, as it is evidenced by the EVEREST study [31], the IN – CHF registry [32] and the ANCHOR study [36]. In our study, at hospital discharge, ACEI were significantly less often prescribed to anemic patients. This might have been due to worse renal function in anemic patients.

HF patients with anemia and without anemia has a significant disparity in the long-term prognosis. Studies conducted by Agata Tyminska et al., Asa Jonsson et al. and the CHARM program, showed anemia has a poor prognostic outcome in patients with heart failure [28, 30, 33]. In the current study, Kaplan Meier survival curve showed (Log Rank test, *P* = 0.042) a significant difference in survival status which is in alignment with the above studies. Further survival curve analysis based on patient’s hemoglobin level indicated an even stronger difference (Log rank test, *P* = 0.001) in the overall mortality in the study group. This result was further supported by findings presented by various studies [29, 35, 37], as lower hemoglobin is strongly related with poor survival outcome.

In the current study, multivariate cox regression analysis showed that the independent factors of all causes of death in patients with HF were age (AHR = 1.041 (1.017 – 1.066), *P* = 0.001), sodium level (AHR = 0.933 (0.882 – 0.987), *P* = 0.016), creatinine level (AHR = 1.869 (1.460 – 2.392), *P* = < 0.0001), and prescription of medications like, ACEI (AHR = 0.410 (0.197–0.852), *P* = 0.017), and Spironolactone (AHR = 0.527 (0.276 – 0.996), *P* = 0.050). Our findings were in line with different studies; in studies investigated by Macín SM. et al. [38], and Ojeda S. et al. [39] in Spain, Agata Tymińska et al. [28] in Poland, Abebe et al. [18] in Ethiopia, a retrospective study in USA by Owan TE et al. [40] showed unfavorable prognosis in HF cohorts who were at advanced age, with lower level of sodium and higher serum creatinine level. Aldosterone blockers had a pivotal advantage in decreasing morbidity and mortality by lowering the atrial natriuretic peptide concentrations [41]. The polish cohort of two European society of cardiology heart failure registries determined that addition of ACEI in HF treatment had significantly decreased mortality in patients with HF [28].

The outcome of our study suggests that although anemia is a strong indicator of unfavorable prognosis in HF, it is not an independent risk factor for adverse outcomes. This may be dictated by the fact that most predictors of anemia, such as older age, higher NYHA class at hospital admission, kidney disease and diabetes overlapped with predictors of clinical endpoints [28].

### Limitation of the study

Our study has several limitations. First, the study was conducted in single center so it might be difficult to represent the nationwide prevalence and prognosis outcome. Second, due to the small sample size, variables which might be statistically significant may not be evident. Third, due to the retrospective nature of the study and the sample size, generalizability to the other center might be taken in caution. Finally, last hospital discharge or medication refill was used to determine time for survival analysis and this could be affected by documentation or loss to follow-up.

Despite these limitations, we believe that our study provides prominent information on the clinical features and prognosis of HF patients with anemia. Moreover, it will provide a blue print for further clinical research in the area.

## Conclusion

In the current study, HF patients with anemia tend to be older age, had lower hemoglobin and sodium level and higher creatinine value. On the other hand, patients without anemia were prescribed ACEI, beta – blocker and spironolactone more frequently than anemic patients. Age, sodium, creatinine and prescription of medication like ACEI and spironolactone predicted all causes of mortality in the study cohorts. Further survival analysis showed there was a significant difference in overall prognosis among anemic and non – anemic, and hemoglobin level. Even though, anemia is a significant risk marker, it is not an independent predictor of mortality in the current study.

## Abbreviations

ACEI: Angiotensin converting enzyme inhibitor
AF: Atrial Fibrillation
BP: Blood pressure
CCB: Calcium channel blocker
CHARM: the Candesartan in Heart Failure Assessment of Reduction in Mortality and Morbidity program
Cr: Creatinine
DCMP: Dilated Cardiomyopathy
GURH: Gondar University Referral Hospital
Hct: Hematocrit
HF: Heart failure
HFpEF: Heart failure with preserved ejection fraction
HFrEF: Heart failure with reduced ejection fraction
Hg: Hemoglobin
HHD: Hypertensive heart disease
IHD: Ischemic Heart Disease
LVEF: Left ventricular ejection fraction
NYHA: New York Heart Association
STAMINAHFP: Study of anemia in a population with heart failure
Val – HeFT: Valsartan heart failure trial
VHD: Valvular Heart Disease
WHO: World health organization
